# Chemical Mixtures in Household Environments: In Silico Predictions and In Vitro Testing of Potential Joint Action on PPARγ in Human Liver Cells

**DOI:** 10.3390/toxics10050199

**Published:** 2022-04-19

**Authors:** Celeste K. Carberry, Toby Turla, Lauren E. Koval, Hadley Hartwell, Rebecca C. Fry, Julia E. Rager

**Affiliations:** 1 Department of Environmental Sciences and Engineering, Gillings School of Global Public Health, The University of North Carolina at Chapel Hill, Chapel Hill, NC 27599, USA; ccarberry@unc.edu (C.K.C.); tobytman@live.unc.edu (T.T.); lkoval@unc.edu (L.E.K.); hadley_hartwell@med.unc.edu (H.H.); rfry@unc.edu (R.C.F.); 2 The Institute for Environmental Health Solutions, Gillings School of Global Public Health, The University of North Carolina at Chapel Hill, Chapel Hill, NC 27599, USA; 3 Curriculum in Toxicology and Environmental Medicine, School of Medicine, University of North Carolina, Chapel Hill, NC 27599, USA

**Keywords:** mixtures, in vitro, toxicology, in silico

## Abstract

There are thousands of chemicals that humans can be exposed to in their everyday environments, the majority of which are currently understudied and lack substantial testing for potential exposure and toxicity. This study aimed to implement in silico methods to characterize the chemicals that co-occur across chemical and product uses in our everyday household environments that also target a common molecular mediator, thus representing understudied mixtures that may exacerbate toxicity in humans. To detail, the Chemical and Products Database (CPDat) was queried to identify which chemicals co-occur across common exposure sources. Chemicals were preselected to include those that target an important mediator of cell health and toxicity, the peroxisome proliferator activated receptor gamma (PPARγ), in liver cells that were identified through query of the ToxCast/Tox21 database. These co-occurring chemicals were thus hypothesized to exert potential joint effects on PPARγ. To test this hypothesis, five commonly co-occurring chemicals (namely, benzyl cinnamate, butyl paraben, decanoic acid, eugenol, and sodium dodecyl sulfate) were tested individually and in combination for changes in the expression of *PPARγ* and its downstream target, insulin receptor (*INSR*), in human liver HepG2 cells. Results showed that these likely co-occurring chemicals in household environments increased both *PPARγ* and *INSR* expression more significantly when the exposures occurred as mixtures vs. as individual chemicals. Future studies will evaluate such chemical combinations across more doses, allowing for further quantification of the types of joint action while leveraging this method of chemical combination prioritization. This study demonstrates the utility of in silico-based methods to identify chemicals that co-occur in the environment for mixtures toxicity testing and highlights relationships between understudied chemicals and changes in PPARγ-associated signaling.

## 1. Introduction

Humans are exposed to numerous chemicals in their everyday environments, predominately in the form of mixtures. However, chemical exposure and toxicity assessments are often designed to evaluate relationships between individual chemical exposures and health outcomes. Commonly investigated chemicals in household environments with known health hazards include parabens, phthalates, plasticizers, flame retardants, and other chemicals that are used in the manufacturing of products and personal care items [1,2,3]. There is currently limited literature on the potential joint toxic effects of chemicals commonly found in everyday household environments on human health. Chemicals can have enhanced effects when exposures occur in the presence of other chemicals that induce similar health outcomes through similar mechanisms of action. A major limitation in mixtures-related exposure and toxicity testing surrounds feasibility, where it is not feasible to test every single possible chemical combination that may occur in the environment. Thus, prioritization and characterization efforts are needed to identify chemical mixtures that are most prevalent in human exposure environments that likely exert toxicity through mechanisms of joint action.

A mechanism through which chemicals can exert joint action is through the targeting of the same molecular mediator(s) that underlie resulting disease outcomes [4,5]. An example of an important molecular mediator of cell health and toxicity is the peroxisome proliferator activated receptor gamma (PPARγ). Previous studies have incorporated mixtures modeling into the evaluation of environmental effects on PPARγ activity and have established PPARγ as an important target for future studies [6]. Peroxisome proliferator activated receptors (PPARs), in general, are a group of nuclear receptor proteins that regulate cell differentiation and metabolic pathways, such as lipid and glucose homeostasis [7]. PPARs regulate gene expression by controlling transcription activity and are present throughout the nuclei of cells [8,9]. The family of PPARs includes three isoforms: PPARα (alpha), PPARβ/δ (beta), and PPARγ (gamma). These three nuclear receptor isoforms are all stimulated by endogenous or exogenous ligands. PPARγ was selected as the potential common molecular mediator of focus for this analysis because of its confirmed status as a major regulator of cell health [7], as well as the availability of PPARγ assay data with adequate response variability from the screening of thousands of chemicals in human liver cells through the ToxCast/Tox21 consortium [10].

PPARγ is expressed throughout several tissues of the body, including adipose, intestinal, kidney, liver, placenta, and spleen tissues [11]. PPARγ, in general, plays important roles in adipogenesis, lipid metabolism, insulin sensitivity, immune regulation, and general cellular processes, including cell differentiation [12]. Increased gene expression of *PPARγ* has been associated with increased PPARγ activity. A recent example study concluded that the expression levels of *PPAR*γ were highly related and co-expressed with important *PPAR*γ gene targets involved in autophagy at various stages of adipocyte differentiation [13]. Furthermore, studies using liver cells have shown that increases in PPARγ activity resulted in altered expression of critical genes, including insulin receptor (*INSR*), sex hormone-binding globulin, glucokinase, and others that are specifically known to be regulated by PPARγ [14,15,16]. Because of its involvement across many cellular processes and associated signaling, altered expression of *PPARγ* has implications in a variety of disease outcomes, including autoimmune disease, cancer, cardiovascular disease, and metabolic disease, among others [17,18,19,20,21,22]. The knowledge of the role of PPARγ specifically in the liver has expanded in recent years, where PPARγ is now recognized as a major regulator of liver metabolism, representing an important function that affects the overall health of organisms [12]. Given the growing literature on PPARγ and its role in a variety of diseases, the continued investigation of this receptor is needed in relation to environmental insults. Here, we expand on this field of study by using database informatics to prioritize which mixtures occur in environmental exposure that may be impacting PPARγ.

The purpose of this study was to couple in silico database mining with in vitro testing to identify chemicals that are likely to co-occur in household environments, and to test their potential joint action on PPARγ in human liver cells as a proof of principal. Chemical exposure information was specifically analyzed from the Chemical and Products Database (CPDat) to identify which chemicals are present across common exposure sources that are relevant to household environments. Chemicals were preselected to include those that increase PPARγ activity in liver cells, and were identified through query of the ToxCast/Tox21 database. The individual vs. joint effects of these chemicals on PPARγ was evaluated on the basis of upstream *PPARγ* expression and a downstream target of PPARγ activity (i.e., *INSR* expression) using HepG2 cells, paralleling the cell type used in ToxCast/Tox21 screening. Findings yielded a novel mixture of understudied chemicals that likely co-occur in household environments and may exacerbate or otherwise influence the toxicological impacts from environmental exposures under conditions of co-exposure. The novelty of this study surrounds the demonstration of in silico modeling to more efficiently identify harmful chemicals and chemical mixtures in the environment which can then be further evaluated through experimental testing.

## 2. Materials and Methods

### 2.1. Organizing Chemical Exposure Data from CPDat

The Chemicals and Products Database (CPDat) is a large, worldwide collection of chemical use inventories and chemical exposure-level data [23,24]. CPDat includes an exposure resource referred to as the Chemical List Presence database, which is organized from manual reviews of federal and state reports, academic journal articles, and publications from international government agencies. Chemical records within this database include global chemical use and product information inventories, chemical safety guideline sheets, food inventories, pesticide use information, and water and soil contamination data. Given the breadth of chemical inventories pulled, this database does not include information on exposure concentrations; rather, it provides information on whether or not a chemical has been recorded as found in a particular product and/or media (i.e., records of absence/presence). This study leveraged data from CPDat for the purpose of identifying possible chemical exposure combinations within household environments that also affect PPARγ pathways. 

The CPDat Chemical List Presence dataset was analyzed, which included chemical and product use information across more than 20,000 chemicals at the time of the analysis (CPDat v2.0) [24]. This dataset contained general chemical use descriptions assigned through “keywords” that have been manually curated to summarize chemical and use categories. We further categorized these keywords into larger “exposure source categories” to allow for improved downstream analyses and exposure pattern recognition. The resulting exposure source categories provide higher-level descriptions of the chemical use and presence information. The specific mapping of CPDat Chemical List Presence keywords to the exposure source categories is provided in the supplemental material (Supplemental Table S1).

### 2.2. Identifying Chemicals That Target PPARγ

This research focused on chemical agonists that target PPARγ in the same target cell type, representing those that may display joint toxicities during conditions of co-exposure. Chemicals were specifically selected to include those likely to increase the activity of PPARγ in HepG2 cells, using findings from the ToxCast/Tox21 in vitro high-throughput screening program [10]. Altered PPARγ activity was prioritized as the molecular endpoint of interest on the basis of the following lines of reasoning: (1) PPARγ is a critical mediator of cell health and toxicity and plays important roles in many human disease outcomes, including metabolic disease and cancer [12]; (2) PPARγ activity endpoints from ToxCast/Tox21 showed data distributions that allowed for the identification of an adequate number of chemicals with assay activity to analyze. Notably, many other endpoints that are relevant to liver biological processes and toxicity (e.g., Hypoxia Inducible Factor 1 Subunit Alpha [HIF1A], and Peroxisome Proliferator Activated Receptor Alpha [PPARα]) did not exhibit as many instances of activity in association with chemicals with exposure information. Therefore, altered PPARγ activity was selected as the focus of the current investigation, which allowed for high data coverage and consistently reported in vitro findings. 

The ToxCast/Tox21 in vitro screening program represents a federal collaboration between the U.S Environmental Protection Agency (U.S. EPA), the National Institutes of Health’s National Toxicology Program and National Center for Advancing Translational Sciences, and the Food and Drug Administration. The ToxCast/Tox21 screening database includes molecular response information across various cell lines treated in dose–response to thousands of individual chemicals, many of which represent understudied chemicals in the environment. This project used publicly available data compiled through the U.S. EPA’s summary-level files (invitrodb_v3.1, released on March 2018) [25]. The analyzed assay endpoint was specifically denoted as the assay_component_endpoint_name of ATG_PPARg_TRANS_up. This endpoint represents a reporter gene assay, which measured mRNA induction to evaluate PPARγ activity at the transcription factor level. Induction of mRNA by PPARγ gene transcription activity was obtained via fluorescent labeling of the PPARγ response element in human HepG2 cells treated in dose–response (largely, 0–1000 µM) to thousands of individual chemicals. Single treatments were carried out for chemicals dissolved in DMSO, and endpoint measures were collected 24 h post-exposure. Given the role of PPRE activity occurring in combination with PPARγ to influence downstream biology [26], ToxCast/Tox21 data were also organized for the assay endpoint, ATG_PPRE_CIS_up, in a similar manner. Raw data were collected and processed by the ToxCast/Tox21 consortium, as previously published [27,28]. 

Here, summary-level data were organized and used to establish filters to identify chemicals that elicited increased PPARγ activity at concentrations outside of those that induce cytotoxicity. Specifically, chemicals were first filtered to include those that were identified as “active” in this assay endpoint, designated as those with hit call values of 1, as previously defined [28]. Then, chemicals were filtered for those that elicited activity at concentrations far below the “cytotoxic signal burst” region, designated as those with cytotoxicity distributions that occurred at standard Z-scores > 2, in comparison to assay bioactivities, as previously defined [27]. These filters parallel those that have previously been implemented when analyzing and interpreting ToxCast/Tox21 data [29,30,31].

### 2.3. Exposure Co-Occurrence Characterization of Environmental Chemicals

Chemicals with exposure information and evidence for inducing PPARγ activity in liver cells were then evaluated for co-occurrence patterns across exposure source categories. Co-occurrence patterns were identified using an approach that coupled standard clustering algorithms with Jaccard similarity indices, based on similarities across exposure source occurrences. The Jaccard index, also known as the Tanimoto index, is a widely used binary distance metric employed in chemoinformatic analyses and applications [32]. Example uses of this metric include the evaluation of chemical fingerprints [33,34] and identification of gene and chemical toxicogenomic profiles [35]. These methods build upon similar clustering approaches that we have previously used to characterize trends across chemical/molecular signatures [36,37].

Here, a summary table of the included chemicals and exposure source categories was organized, containing values of 1, which indicated chemicals that were present in an exposure source category, and values of 0, which indicated chemicals that were not present in an exposure source category. This summary table was then used to calculate a distance matrix based upon the Jaccard distance measures, which represent the complement of the Jaccard similarity indices [38]. Values within the resulting distance matrix can range from 0, representing low dissimilarity (i.e., high similarity), to 1, representing high dissimilarity (i.e., low similarity). The vegan package (v2.5.7) was used to carry out these calculations in R (v4.0.3). These final Jaccard distance values were used as input towards a hierarchical clustering analysis. In determining the number of clusters to use, the average silhouette width and within cluster sum of squares of the distance matrix were derived, and the results were visualized across 1≤ k ≤ 34 clusters through the factoextra package in R (v1.0.7). Data were then grouped into the optimized number of clusters using the cluster package (v2.1.1). Parallel methods were used to determine the clusters of exposure source categories. In visualizing these clustering results, a heatmap was generated using the pheatmap package (v1.0.12). 

### 2.4. Selection of High-Interest Chemicals That Co-Occur as Mixtures for In Vitro Testing

The chemical exposure pattern analysis findings were used to prioritize a cluster of chemicals that exhibited similar co-occurrence patterns across exposure source categories. One cluster was identified as high interest due to the following lines of reasoning: (1) This cluster included chemicals that were present across the highest number of exposure source categories, on average; (2) Chemicals in this cluster have many recorded instances of being present in exposure source categories that are relevant to our everyday household environments. Because this cluster contained nine chemicals, we further informed the final selection of chemicals to carry forward for in vitro testing to cover various product uses for chemicals that were also available to purchase from credible chemical vendors. 

### 2.5. Chemical Procurement for In Vitro Testing

The five chemicals that were prioritized for in vitro testing were purchased from the following chemical suppliers: Butylparaben was purchased from Alfa Aesar (Haverhill, MA, USA; Cat: A14043, Purity of 99+%); benzyl cinnamate from Alfa Aesar (Haverhill, MA, USA, Cat: A19550, Purity of 99%); Decanoic acid from Alfa Aesar (Haverhill, MA, USA, Cat: A14788, Purity of 99%); Eugenol from Alfa Aesar (Haverhill, MA, USA, Cat: A14332, Purity of 99%); and Sodium dodecyl sulfate (SDS) from TCI America (Portland, Oregon, Cat: I035225G, Purity of 97+%). Chemicals were dissolved in DMSO to generate high-concentration stock solutions that were further diluted in cell-culture media for final treatment conditions, as detailed below. 

### 2.6. Cell Culture and Treatment

The HepG2 immortalized human liver cell line was purchased from Sigma Aldrich (St. Louis, MO, USA). The cells were grown in Gibco Minimum Essential Media supplemented with 10% fetal bovine serum at 37 °C in 5% carbon dioxide. Cells were plated at 2 × 10^6^ cells per 100 mm dish and incubated under standard conditions until achieving 70–80% confluence. To investigate the cytotoxicity of the evaluated chemicals in vitro, HepG2 cells were seeded in a 96-well culture plate at 10^4^ cells per well and incubated for 24 h prior to treatment. Similarly, to investigate *PPARγ* and *INSR* expression in vitro, HepG2 cells were seeded in a 12-well culture plate at 10^5^ cells per well and incubated for 24 h prior to treatment. On the day of treatments, stock chemical solutions dissolved in DMSO were added to cell culture medium and vortexed to create final concentrations. Chemical treatments or vehicle controls (i.e., DMSO added to cell culture media) were added to cells, incubated for 24 h, and harvested for downstream analysis. More details are provided per assay-specific methods below.

### 2.7. Cytotoxicity Assay

For downstream experiments, an activity concentration where 90% viability is achieved (AC_90_) needed to be established to observe bioactivity in the absence of excess cell death. To establish the AC_90_ for each chemical, HepG2 cells seeded in 96-well plates were treated in triplicate with concentrations of benzyl cinnamate, butylparaben, decanoic acid, eugenol, and sodium dodecyl sulfate ranging from 0 to 2000 μM to include concentrations implemented within ToxCast/Tox21. Chemical treatments were incubated for 24 h, followed by the addition of resazurin, according to the manufacturer’s protocol. In brief, resazurin was dissolved in filtered Dulbecco’s phosphate-buffered saline to a concentration of 15 mg/kg and added to each well. Cells were incubated for 2 h at 37 °C in 5% CO_2_. Fluorescence was read on a SpectraMax iD5 Multi-Mode Microplate Reader (Molecular Devices, San Jose, CA, USA) using 560 and 590 nm as the excitation and emission wavelength, respectively. Dose–response cytotoxicity curves were generated using a nonlinear regression model within GraphPad Prism (v9.0) to estimate each AC_90_ to carry forward in the in vitro testing. In one case, a chemical was not found to decrease viability to 90% at concentrations up to the maximum tested (2000 μM), and a concentration of 1000 μM was carried forward in the analysis of this chemical. Descriptive statistics, including mean and standard deviation, were calculated within GraphPad Prism and plotted on dose–response curves. The same methods were used to assess cell viability in response to mixtures exposures. These mixtures included all five chemicals tested at concentrations that were proportional to their respective AC_90_. The following mixtures concentrations were specifically tested: C_1.0_, C_0.5_, and C_0.2_, where C_i_ was defined through the following Equation (1):

(1) $$\begin{array}{c} C_{i}=\left({\mathrm{AC}}_{90,\;\mathrm{Benzyl}\;\mathrm{Cinnamate}}\times \;i\right)+\left({\mathrm{AC}}_{90,\;\mathrm{Butyl}\;\mathrm{Paraben}}\times \;i\right)+\left({\mathrm{AC}}_{90,\;\mathrm{Decanoic}\;\mathrm{Acid}}\times \;i\right)\\ +\left({\mathrm{AC}}_{90,\;\mathrm{Eugenol}}\times \;i\right)+\left({\mathrm{AC}}_{90,\;\mathrm{SDS}}\times \;i\right) \end{array}$$

for i = 1.0, 0.5, and 0.2.

### 2.8. PPARγ and INSR Gene Expression Screening

Given the relationship between *PPARγ* gene expression and PPARγ transcription factor activity, *PPARγ* gene expression was measured as an indicator of PPARγ-associated signaling [13]. To further evaluate PPARγ activity, verified gene targets of PPARγ transcription factor activation were queried within the PPARgene database, where *INSR* was identified as a validated target in HepG2 cells [26]. Gene expression of *INSR* was thus selected for measurement. To examine *PPARγ* and *INSR* gene expression, HepG2 cells seeded in 12-well plates were treated with chemical concentrations summarized in Table 1. 

After 24 h incubation, treated and vehicle control cells were collected in 350 μL of Qiagen Buffer RLT Plus for RNA extraction using the Qiagen RNeasy Mini Kit, according to the manufacturer’s protocol, and RNA samples were quantified using a Nanodrop 1000 spectrophotometer (Thermo Scientific, Waltham, MA, USA). Exposure conditions were tested in biological triplicate. To analyze gene expression, extracted RNA was converted to cDNA using the High-Capacity cDNA Reverse Transcription kit (Applied Biosystems, Foster City, CA, USA). Real-Time quantitative reverse transcription polymerase chain reaction (RT-qPCR) was performed in biological and technical triplicate using Qiagen’s RT-qPCR Primer Assay for *PPARγ* (cat. 330001, GeneGlobe ID: PPH02291G) and *INSR* (cat. 330001, GeneGlobe ID: PPH02324F). Expression values based on cycle thresholds (Cts) were measured using a Stratagene Mx3000P instrument (Agilent, Santa Clara, CA, USA). Values were normalized against the geometric mean of the housekeeping gene, glyceraldehyde 3-phosphate dehydrogenase (*GAPDH*), and fold changes in expression were calculated using the ddCt method [39]. Data were visualized using GraphPad Prism.

## 3. Results

### 3.1. Study Overview

**Figure 1:**
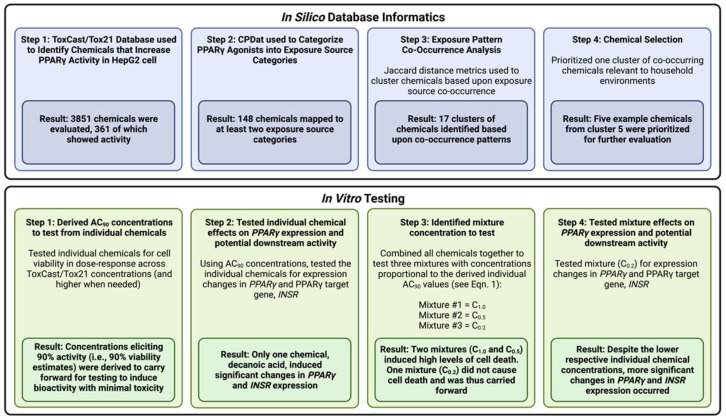
**Study experimental workflow.** This figure illustrates the experimental workflow, from in silico prioritization and characterization of chemicals to in vitro testing. Figure created in BioRender.

An overview of the steps carried out in this project is illustrated in Figure 1. In brief, database mining and in silico methods were used to prioritize chemicals that are likely to co-occur in our everyday household environments as mixtures exposures that also commonly target PPARγ. In silico approaches were specifically used to: (1) identify chemicals that increase PPARγ activity in HepG2 cells; (2) categorize PPARγ agonists into exposure source categories; (3) evaluate chemical co-occurrence patterns relevant to household environments; and (4) prioritize chemicals for in vitro analysis. These in silico database informatic approaches informed a proof-of-principal in vitro study in a parallel HepG2 model. In brief, selected chemicals were: (1) tested individually to derive AC_90_ concentrations; (2) tested individually for changes in the expression of *PPARγ*, as well as its target gene, *INSR*, as a measure of potential downstream activity; (3) combined within mixtures for concentration selection; and (4) tested as a mixture to evaluate changes in expression of *PPARγ*, as well as its target gene, *INSR*, as a measure of potential downstream activity. Results highlight the utility of merging in silico database informatics with in vitro testing to evaluate environmental mixtures.

### 3.2. Identification of Chemicals That Increase PPARγ Activity in Human Liver Cells

This analysis focused on chemicals that impact a common molecular mediator that is known to play a critical role in liver biological processes and toxicity, PPARγ. In identifying these chemicals, data from the ToxCast/Tox21 in vitro high-throughput screening program (v3.1) were analyzed to capture understudied chemicals of environmental relevance. Within this screening effort, a total of 3851 chemicals were evaluated for their potential to induce changes in PPARγ activity in human liver HepG2 cells. Of these, 364 were shown to cause increased PPARγ activity at concentrations well below cytotoxicity. Data surrounding the ToxCast/Tox21 PPARγ assay results, including detailed chemical identifiers, hit calls, cytotoxicity z-scores, and AC_50_ values, are detailed in Supplemental Table S2.

### 3.3. Dataset of Chemicals with Exposure Information and Evidence of PPARγ Activity Changes

This analysis focused on chemicals with demonstrated evidence of increasing the expression of *PPARγ* in human liver HepG2 cells that also contained exposure data from CPDat mapping to exposure source categories. Here, exposure source categories represent high-level summaries of keywords that describe chemical and product use information derived through chemical inventory information contained within CPDat, in the form of recorded absence/presence data. Chemical data were filtered for chemicals that were associated with at least two distinct exposure source categories to allow for the analysis of chemical co-occurrence patterns. After these filters were applied, a total of 148 chemicals remained for analysis. These 148 chemicals mapped to 30 exposure source categories across 117 keywords (Supplemental Tables S1 and S3). In addition, chemicals were considered for PPRE agonism, as detailed in Supplemental Table S3.

### 3.4. Characterization of Co-Occurring Chemicals in the Environment That Increase PPARγ Activity

**Figure 2:**
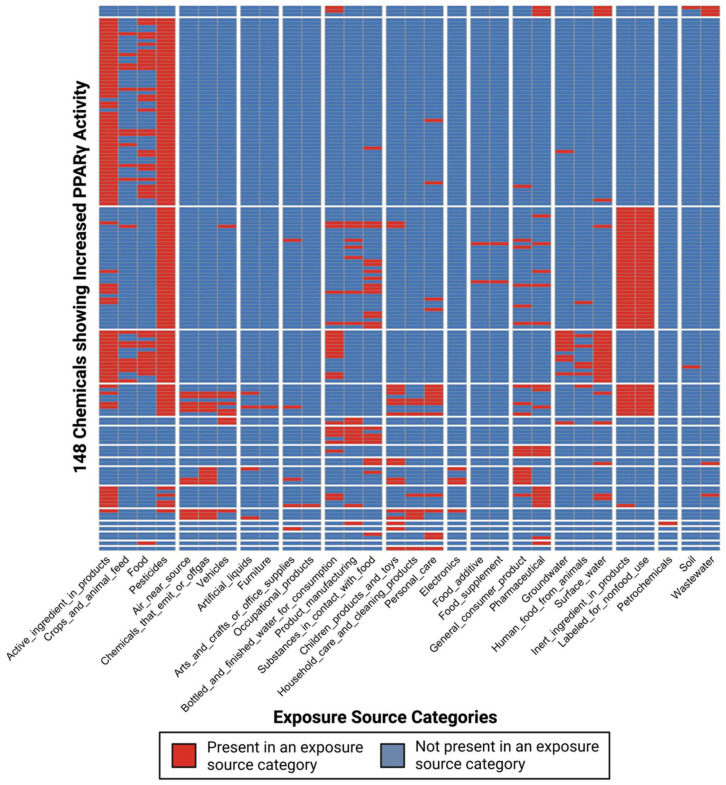
**Clusters of chemicals with evidence of increasing PPARγ activity, arranged based upon co-occurrence patterns across exposure source categories.** Each row reflects a chemical and each column reflects an exposure source category derived through analysis of CPDat. Records of a chemical being present in an exposure source category are shown in red. Chemicals without a recorded instance of being in an exposure source category are shown in blue. Chemical clusters are numbered top to bottom (1–17) and represent chemical mixtures that are likely to co-occur in the environment based on analysis of worldwide chemical and product use information.

Chemicals that contained exposure data and evidence for increasing PPARγ activity in liver cells were evaluated for potential co-occurrence as chemical mixtures that are present across overlapping exposure source categories. A clustering approach was used to derive 17 distinct clusters across the 148 total chemicals, with each cluster containing groups of chemicals with overlapping exposure source categories (Figure 2). These clusters of chemicals represent chemical mixtures that are likely to co-occur in the environment based on analysis of worldwide chemical and product use information. Exposure source categories notably grouped into 13 distinct clusters based on similarly occurring chemicals, representing common sources of exposure that may lead to aggregate exposures of chemicals that co-occur in the environment (Figure 2).

### 3.5. Selection of High-Interest Household Chemicals That Co-Occur as Mixtures for In Vitro Testing

**Figure 3:**
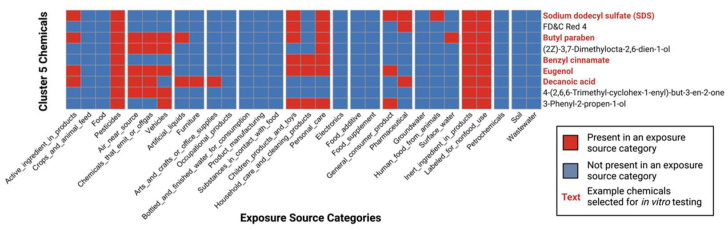
**Prioritized cluster that contained co-occurring chemicals relevant to everyday household environments.** Chemicals in Cluster 5 had recorded instances of being present in household-environment-related exposure source categories, including those that were relevant to food and water (e.g., pesticides, human food from animals, surface water), product off-gassing (e.g., chemicals that emit or off-gas), and household products and materials (e.g., furniture, household care and cleaning products, personal care, and inert ingredient in products). This cluster was prioritized for further evaluation, where five chemicals, indicated by red text, were identified for mixtures-based testing in vitro.

Results from the chemical-exposure-pattern analysis were used to select chemicals of interest to test further via in vitro methods. A cluster of chemicals was prioritized that exhibited similar co-occurrence patterns across exposure source categories; specifically, Cluster 5 (Figure 3). 

This cluster was prioritized based upon the following lines of reasoning: First, this cluster included chemicals that were present across the highest number of exposure source categories, on average. To detail, Cluster 5 contained chemicals with reported presence across an average of eight exposure sources, where the other clusters contained chemicals with reported presence across an overall average of four exposure sources (min = 2; max = 8) (Supplemental Table S4). Second, chemicals within Cluster 5 showed many recorded instances of being present in exposure source categories that are relevant to everyday household environments. These categories included select food- and water-relevant exposure source categories (e.g., pesticides, human food from animals, surface water), exposure source categories from chemicals off-gassing from products (e.g., chemicals that emit or off-gas), and many exposure source categories from household products and materials, including furniture, household care and cleaning products, personal care products, and inert ingredient in products (Figure 3, Supplemental Table S3).

Cluster 5 contained nine chemicals, and a subset of these were carried forward for in vitro testing. This chemical subset was selected to cover variable product uses, while also including those that were able to be purchased, were accessible at the time of research, and were remain stable in solution based upon their physicochemical properties. This selection resulted in a final list of five chemicals that were evaluated further through in vitro testing (Figure 2). In addition to PPARy agonism, four of these prioritized chemicals have also been shown to agonize PPRE (Supplemental Table S3). 

### 3.6. Cell Viability in Response to Treatment Conditions

**Figure 4:**
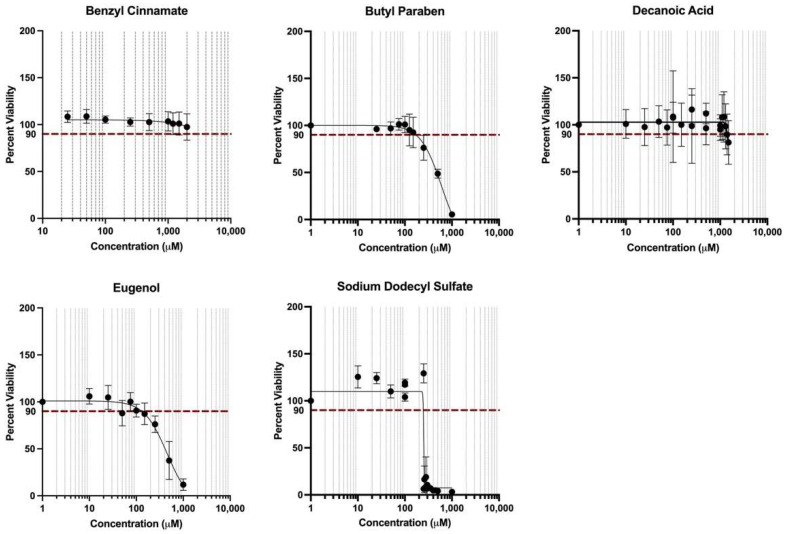
**Cell viability curves generated from cytotoxicity screening of individual chemicals across multiple doses to derive AC_90_ values.** Household chemicals elicited variable cytotoxicity when tested across a range of doses. Mean and standard deviation are plotted for each dose. Predicted dose eliciting 90% viability (AC_90_) is indicated by the red dotted line.

HepG2 cells were treated across various doses to either benzyl cinnamate, butyl paraben, decanoic acid, eugenol, or sodium dodecyl sulfate, and demonstrated variable cytotoxicity as detailed in Figure 4.

Dose–response cytotoxicity curves were used to estimate AC_90_ exposure concentrations, which are summarized in Table 1. These doses were carried forward individually or were proportionally scaled down in the following mixtures experiments. Note that benzyl cinnamate was not found to decrease viability to 90% at concentrations up to the maximum tested (2000 μM), and a concentration of 1000 μM was carried forward in the analysis of this chemical (proportionally scaled down in the mixtures evaluation).

For the mixtures evaluations, tested chemical combinations were summarized with the following abbreviation: C_i_, defined as the sum of [each individual chemical AC_90_] × i. Three combinations of concentrations were tested for viability: C_1.0_, C_0.5_, and C_0.2_, and the resulting viabilities were 12%, 17%, and 136%, on average, respectively. The mixture that elicited <90% viability (i.e., C_0.2_) was carried forward in the following analyses, and the composition for this tested mixture is fully detailed in Table 1. 

### 3.7. PPARγ and INSR Expression Changes in Response to Individual Household Chemicals vs. Household Chemical Mixture

**Figure 5:**
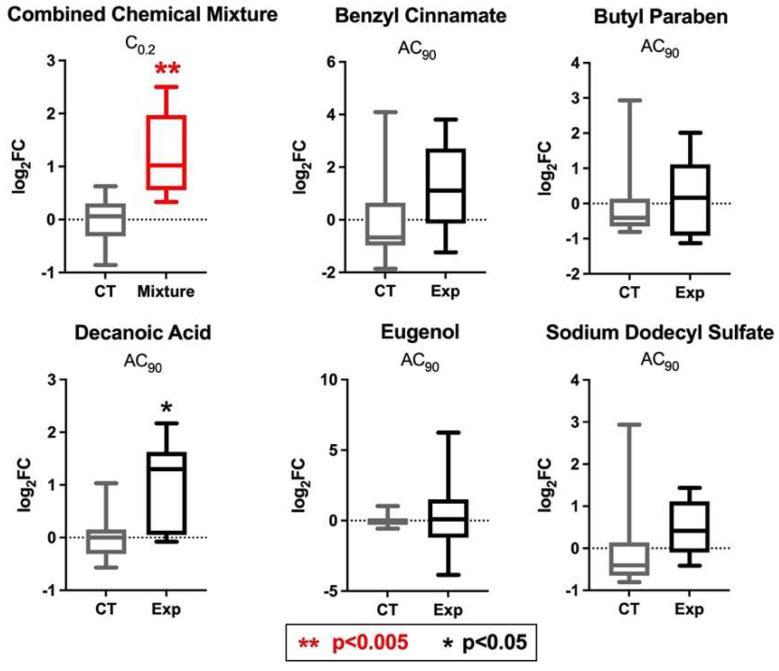
**Changes in *PPARγ* expression in liver cells after exposure to individual chemicals vs. a mixture of chemicals found in exposure sources relevant to everyday household environments.** qRT-PCR results representing the minimum, lower quartile, median, upper quartile, and maximum log_2_ fold changes of *PPARγ* expression associated with individual and combined chemical exposures.

To evaluate whether these co-occurring chemicals potentially exert joint effects on PPARγ-associated signaling, qRT-PCR was used to evaluate potential changes in expression of *PPARγ,* as well as a PPARγ-target gene, *INSR*, in HepG2 cells exposed to individual household chemicals as well as the household chemical mixture. Of the five individual household chemicals tested, all showed modest increases in *PPARγ* expression compared to the control. To detail, all but one of the log_2_ fold changes (log_2_FC) (based off 2^−ddCt^ calculations comparing exposed versus unexposed samples) averaged between 0.22 and 0.94, with one chemical, benzyl cinnamate, reaching a log_2_FC of 1.24, which was not statistically significant compared to the controls. Of all the individual chemicals tested, only one was significant at *p* < 0.05. Specifically, decanoic acid caused *PPARγ* expression to increase at a log_2_FC of 0.94 (*p* = 0.012) (Figure 5).

In comparison, a mixture of all chemicals tested at a concentration 0.2 times that of the doses used for individual testing (i.e., C_0.2_) elicited 0% cytotoxicity and induced a more significant change, with a large fold change in comparison to the individual chemicals. Specifically, this combined mixture caused PPARγ expression to increase at a log_2_FC of 1.2 (*p* = 0.001), with a more significant change than any of the individual chemical test results. These findings support the potential joint action of these chemicals on *PPARγ* expression, highlighting mixtures-induced changes that were amplified in comparison to individual chemical-induced changes.

**Figure 6:**
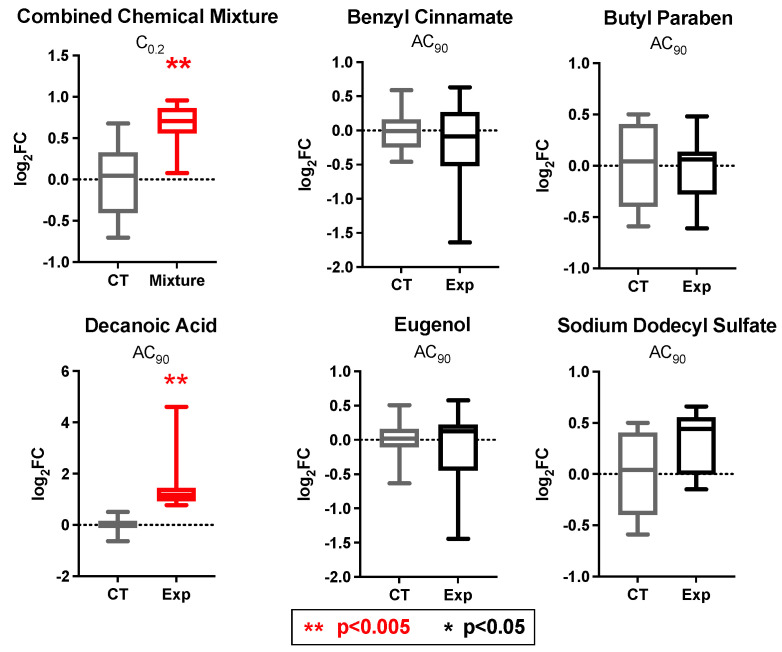
**Changes in *INSR* expression in liver cells after exposure to individual chemicals vs. a mixture of chemicals found in exposure sources relevant to everyday household environments.** qRT-PCR results for *INSR* represent the minimum, lower quartile, median, upper quartile, and maximum log_2_ fold changes of *INSR* expression associated with individual and combined chemical exposures.

Similarly, to evaluate whether these co-occurring chemicals potentially exert joint effects on downstream PPARγ activity, qRT-PCR was used to evaluate *INSR* expression as a verified target gene [26]. HepG2 cells were exposed to household chemicals individually and in a mixture, as described above. Of the five individual household chemicals tested, all showed modest changes in *INSR* expression compared to the control. Specifically, the average log_2_FC ranged from −0.19 to 1.52. Parallel to the evaluation of *PPARγ* expression, only decanoic acid induced statistically significant expression changes, causing an increase at a log_2_FC of 1.52 (*p* = 0.002) (Figure 6).

Additionally, the mixture of all chemicals combined (C_0.2_) induced an increase in *INSR* expression at a log_2_FC of 0.67 (*p* = 0.001). Similar to the *PPARγ* expression analysis, *INSR* expression was increased more significantly by the C_0.2_ mixture than by individual chemicals, even at lower respective concentrations. While decanoic acid appears to be a major driver of *INSR* expression changes, given the concentrations tested, one would expect a relatively lower fold change from the mixture exposure if chemical relationships showed no interactions. However, there is a high significance at a greater fold change induction than expected, based on individual chemical testing. These findings thus support the potential joint action of these chemicals on PPARγ and *INSR* expression, which are indicators of PPARγ activity.

## 4. Discussion

This study represents a novel integration of in silico database informatics with follow-up in vitro testing to evaluate understudied chemicals in our everyday household environments that exert changes on a common molecular mediator, PPARγ. This research resulted in the following major findings: First, we demonstrated the utility of employing informatics and computational methods across the large bioactivity and exposure databases of ToxCast/Tox21 and CPDat, respectively, to identify co-occurring chemicals that likely elicit biological changes through similar mechanisms involving PPARγ. Second, understudied chemicals in household-relevant exposure source categories were prioritized for further evaluation, including those used in personal care products, children’s products and toys, household care and cleaning products, and other ingredients in household products. Third, in vitro testing of five prioritized chemicals demonstrated that individual chemicals exerted less significant changes in expression of *PPARγ* and *INSR*, indicators of PPARγ activity, than a combined exposure of these same chemicals at lower concentrations, demonstrating potential joint effects of these household chemicals in conditions of co-exposure. These common household chemicals may therefore exacerbate exposure-induced effects when exposures occur as mixtures.

Informatics-based approaches were implemented here to predict chemicals that co-occur in the environment as common exposure sources that may be impacting human health. Our approaches leveraged the bioactivity and exposure databases of ToxCast/Tox21 and CPDat, respectively, and implemented high-throughput assay filtering followed by chemical pattern analyses. Pattern analyses leveraged Jaccard similarity indexes acrosschemical use categories with clustering algorithms to yield groups (i.e., clusters) of chemicals that are likely to co-occur in the environment. This approach represents one of many different combinations of in silico approaches that can be leveraged to better understand toxicity and exposure patterns. For example, data reduction approaches have been used to evaluate similarities amongst chemical and biological response profiles associated with complex dietary supplement exposures [40,41]. Random forest modeling approaches have been used leveraging Tox21 high-throughput toxicity screening assays that demonstrated co-occurring activities to predict in vivo apical toxicity outcomes in the rat liver [31]. In terms of human exposure pattern modeling, a recent notable study analyzed chemical biomonitoring data within the National Health and Nutrition Examination Survey (NHANES) through frequent itemset mining, which yielded novel chemical combinations detected to frequently co-occur in humans [42]. Other advanced in silico methods have included additional machine-learning approaches (i.e., deep learning) [43], Bayesian statistical methods [44], and text mining [45]. The current study, as well as these additional examples of silico/database-driven approaches, collectively represent progress in the fields of exposure science and toxicology towards better characterizing environmental mixtures.

The chemicals prioritized for experimental evaluation of individual vs. mixtures-induced impact on PPARγ activity included benzyl cinnamate, butyl paraben, decanoic acid, eugenol, and sodium dodecyl sulfate. These chemicals have evidence to support their presence in children’s products and toys, household care and cleaning products, and personal care products, as curated through CPDat. Benzyl cinnamate is an ester compound derived from cinnamic acid and benzyl alcohol and is used as a scent, flavoring agent, fixative, antibacterial agent, and antifungal agent in certain products, foods, and pharmaceuticals [46]. Butyl paraben (also referred to as 4-hydroxybenzoic acid butyl ester or butyl p-hydroxybenzoate) is synthetically generated and is used as a preservative in cosmetics and food [47]. Decanoic acid (also referred to as capric acid) is naturally present in certain foods, is organically synthesized from esters, and is found primarily in perfumes, artificial fruit flavors, and food additives [48,49]. Eugenol is a chain-derived molecule that is used as a flavoring agent in teas, meats, perfumes, and essential oils [50]. Sodium dodecyl sulfate (also referred to as sodium lauryl sulfate) is an organosulfate compound that is mainly used in cleaning products, such as laundry detergent, because of its properties as an anionic surfactant [51]. While these chemicals may have been originally tested/evaluated for safety at the exposure doses that occur in household items, what remains to be established are the effects of these chemicals in conditions of co-exposure.

This study compared the effects of each individual chemical vs. a mixture of these chemicals on PPARγ activity via measures of upstream *PPARγ* expression and downstream *INSR* expression in HepG2 liver cells. Each chemical was first tested individually at varying concentrations, and many chemicals were found to cause no effect or slightly increased cell viability at lower doses, and decreased cell viability at higher doses. This trend parallels some literature supporting the potential beneficial/protective effects produced by the tested chemicals at lower doses, including benzyl cinnamate [52], butyl paraben [53], decanoic acid [54], and eugenol [55]. At higher doses, there is also evidence of certain chemicals causing liver toxicity, among other toxicological outcomes, including butyl paraben [56], eugenol [55], and sodium dodecyl sulphate [57,58,59]. Our in vitro cell viability results were then used to estimate the chemical concentrations eliciting approximately 90% cell viability to carry forward in the testing of *PPARγ* and *INSR* expression changes, which were then compared to a mixture exposure containing all five of these chemicals. Collectively, this mixtures-based exposure condition elicited a more robust and significant increase in *PPARγ* expression in comparison to any of the chemicals that were tested individually, despite this mixture containing much lower concentrations of each of the individual chemicals. Similarly, *INSR* expression was more significantly altered by the mixture than by the individual chemicals. These data suggest that conditions of co-exposure for these chemicals may exacerbate biological changes occurring in the liver, in comparison to single-chemical exposure conditions. While the doses tested in this study are likely higher than those that reach the liver following exposure in our everyday environment, these data provide important preliminary information towards identifying chemicals that may induce joint toxicity.

Our study employed a novel approach to prioritize and test biological responses associated with chemical mixtures in the environment and serves as a starting point for future investigations that could expand upon these initial findings. We used the ToxCast/Tox21 database to prioritize chemicals that were found previously to increase PPARγ activity in human liver HepG2 cells as a consistent metric that we could then test in our laboratory. It is notable that other biological response databases can be queried to expand upon molecular targets and endpoints of interest, including the Chemical Effects in Biological Systems [60], Comparative Toxicogenomics Database [61], and Toxicological Reference Database [62], among others. We also focused on exposure information gathered through the Chemical List Presence dataset within CPDat, which represents a robust chemical inventory database that captures information across tens of thousands of chemicals. The CPDat’s Chemical List Presence dataset was selected for use because of its wide chemical coverage, as well as its organizational structure on a per-chemical basis. These properties allowed for the analysis of chemicals with both exposure and bioactivity data from CPDat and ToxCast/Tox21, respectively. Future efforts could incorporate additional datastreams that inform human exposure patterns. For example, a recent study used consumer product purchasing data collected through The Nielsen Company and linked these data with available CPDat chemical composition data from SDS/MSDS, Manufacturer Ingredient Disclosures, and Ingredient Lists to understand the composition and function of chemicals in consumer products [63]. These types of data could be integrated in future analyses for more product use based exposure predictions, and could also inform exposure dosings, though will likely limit the scope of chemicals with available data. Future efforts could also incorporate chemical fate and transport mechanisms to enhance the consideration of exposure routes and human intake/dosing through additional databases and/or model evaluations. Additional exposure databases that provide more quantitative measures of exposure could include biological or environmental monitoring data (e.g., NHANES [64], or the Air Quality Survey (AQS) [65]), and additional models could include the Systematic Empirical Evaluation of Models [SEEM] framework [66], among other databases/models.

In terms of in vitro mixtures testing, this study served as a proof-of-principal, providing preliminary evidence of joint effects between the evaluated household chemicals on PPARγ activity through upstream and downstream gene expression in liver cells. Given that multiple mechanisms of toxicity may be at play, future studies may further evaluate these chemicals using -omic technologies to more fully characterize mechanisms involved. Additionally, future studies could more comprehensively evaluate these individual chemicals and defined mixtures, capturing expression changes in dose–response, and conducting mixtures modeling to quantify additivities and/or synergies on *PPARγ* expression and other upstream/downstream targets. Additional studies could also implement similar methods to identify chemicals that may influence other important molecules involved in the regulation of cell health and biology that may be impacted by mixtures exposures.

## 5. Conclusions

In conclusion, this study highlights a novel method of database integration, combined with informatics, to characterize combinations of chemicals that likely occur in the environment as mixtures exposures that also show potential for eliciting biological changes through common molecular mediators. This was exemplified through a case study that focused on PPARγ in human liver cells, and through exposure patterns that were informed by an analysis of the CPDat database. Results yielded findings on co-occurring chemicals in household environments that increased expression of *PPARγ* and its target gene, *INSR*, to a greater extent when exposures occurred as mixtures. Future studies will further quantify the relationships between these potential joint toxicities while expanding the prioritization efforts towards additional biological targets and chemical exposure domains.

## Figures and Tables

**Table 1:** The AC_90_ concentrations of individual household chemicals and a household chemical mixture used to evaluate exposure-induced changes in *PPARγ* expression and *INSR* expression in HepG2 cells. The resulting viabilities of each evaluated condition are also provided. Note that higher concentrations of chemicals were tested as a combined mixture, but caused a sudden, significant drop in viability at doses higher than C_0.2_.

Chemical Name	CASRN	Individual Chemical Concentrations Tested (AC_90_) (μM)	Concentrations of Chemicals Tested as a Mixture (at C_0.2_) ^1^ (μM)
Benzyl cinnamate	103-41-3	1000	200
Butylparaben	94-26-8	150	30
Decanoic acid	334-48-5	1300	260
Eugenol	97-53-0	150	30
Sodium Dodecyl Sulfate	151-21-3	250	50
% Viability of each treatment ^2^		90%	136%

^1^ C_0.2_ was defined as the sum of each individual chemical eliciting 90% viability × 0.2. Therefore, each chemical was included in the mixture at concentrations that were 1/5 of their respective AC_90_ values. ^2^ Benzyl cinnamate did not decrease viability to 90% in any of the concentrations tested and was thus at 100% viability at the tested concentration.

## Data Availability

All data generated and analyzed in this study were organized to incorporate Findability, Accessibility, Interoperability, and Reusability (FAIR) principals [67]. CPDat and ToxCast/Tox21 data are publicly available [25]. Script and associated data that were used in the analysis of the exposure patterns and the resulting visualizations are provided online through the Ragerlab Github repository [68]. Newly generated data from the in vitro testing of individual chemicals and the chemical mixtures induced impacts on *PPARγ* and *INSR* expression are published through the UNC Dataverse, and specifically on the Ragerlab-Dataverse repository [69].

## Supplementary Materials

The following are available online at https://www.mdpi.com/article/10.3390/toxics10050199/s1, Table S1: Data used to evaluate human exposure patterns through the organization of exposure source categories; Table S2: Chemicals that induced an increase in the expression of PPARg in HepG2 cells from the ToxCast/Tox21 high-throughput screening program; Table S3: Database of 148 chemicals and their associated exposure source categories; Table S4: Average number of instances chemicals were present in an exposure source category, calculated on a per-cluster basis.

## References

[1] Rager J.E., Strynar M., Liang S., McMahen R.L., Richard A.M., Grulke C., Wambaugh J., Isaacs K., Judson R., Williams A. (2016). Linking high resolution mass spectrometry data with exposure and toxicity forecasts to advance high-throughput environmental monitoring. Environ. Int..

[2] Hoffman K., Garantziotis S., Birnbaum L., Stapleton H.M. (2015). Monitoring Indoor Exposure to Organophosphate Flame Retardants: Hand Wipes and House Dust. Environ. Health Perspect..

[3] Guo Y., Kannan K. (2013). A Survey of Phthalates and Parabens in Personal Care Products from the United States and Its Implications for Human Exposure. Environ. Sci. Technol..

[4] Rider C., Furr J.R., Wilson V., Gray L.E. (2010). Cumulative effects of in utero administration of mixtures of reproductive toxicants that disrupt common target tissues via diverse mechanisms of toxicity. Int. J. Androl..

[5] Rider C.V., Carlin D.J., DeVito M.J., Thompson C.L., Walker N.J. (2013). Mixtures research at NIEHS: An evolving program. Toxicology.

[6] Watt J., Webster T.F., Schlezinger J.J. (2016). Generalized Concentration Addition Modeling Predicts Mixture Effects of Environmental PPARγ Agonists. Toxicol. Sci..

[7] Grygiel-Górniak B. (2014). Peroxisome proliferator-activated receptors and their ligands: Nutritional and clinical implications—A review. Nutr. J..

[8] Choi J.-M., Bothwell A.L.M. (2012). The nuclear receptor PPARs as important regulators of T-cell functions and autoimmune diseases. Mol. Cells.

[9] Patel H., Truant R., Rachubinski R.A., Capone J.P. (2005). Activity and subcellular compartmentalization of peroxisome proliferator-activated receptor α are altered by the centrosome-associated protein CAP350. J. Cell Sci..

[10] Toxicology Testing in the 21st Century (Tox21)|Safer Chemicals Research|US EPA. https://www.epa.gov/chemical-research/toxicology-testing-21st-century-tox21.

[11] NCBI (2021). PPARG Peroxisome Proliferator Activated Receptor Gamma. https://www.ncbi.nlm.nih.gov/gene/5468.

[12] Wang Y., Nakajima T., Gonzalez F.J., Tanaka N. (2020). PPARs as Metabolic Regulators in the Liver: Lessons from Liver-Specific PPAR-Null Mice. Int. J. Mol. Sci..

[13] Ahmed M., Lai T.H., Hwang J.S., Zada S., Pham T.M., Kim D.R. (2019). Transcriptional Regulation of Autophagy Genes via Stage-Specific Activation of CEBPB and PPARG during Adipogenesis: A Systematic Study Using Public Gene Expression and Transcription Factor Binding Datasets. Cells.

[14] Kim T.-H., Kim H., Park J.-M., Im S.-S., Bae J.-S., Kim M.-Y., Yoon H.-G.Y., Cha J.-Y., Kim K.-S., Ahn Y.H. (2009). Interrelationship between Liver X Receptor α, Sterol Regulatory Element-binding Protein-1c, Peroxisome Proliferator-activated Receptor γ, and Small Heterodimer Partner in the Transcriptional Regulation of Glucokinase Gene Expression in Liver. J. Biol. Chem..

[15] Costa V., Foti D., Paonessa F., Chiefari E., Palaia L., Brunetti G., Gulletta E., Fusco A., Brunetti A. (2008). The insulin receptor: A new anticancer target for peroxisome proliferator-activated receptor-g (PPARg) and thiazolidinedione-PPARg agonists. Endocr.-Relat. Cancer.

[16] Selva D.M., Hammond G. (2009). Peroxisome-Proliferator Receptor γ Represses Hepatic Sex Hormone-Binding Globulin Expression. Endocrinology.

[17] Chi C.-W., Hsu H.-T. (2014). Emerging role of the peroxisome proliferator-activated receptor-gamma in hepatocellular carcinoma. J. Hepatocell. Carcinoma.

[18] Di Marzio D. (2008). Peroxisome proliferator-activated receptor-γ agonists and diabetes: Current evidence and future perspectives. Vasc. Health Risk Manag..

[19] Motawi T.K., Shaker O.G., Ismail M.F., Sayed N.H. (2017). Peroxisome Proliferator-Activated Receptor Gamma in Obesity and Colorectal Cancer: The Role of Epigenetics. Sci. Rep..

[20] González-Castro T.B., Tovilla-Zárate C.A., Juárez-Rojop I.E., Hernández-Díaz Y., López-Narváez M.L., Rodríguez-Pérez C., González-Hernández Y.K., Ramos-Méndez M. (2018). Ángel PON2 and PPARG polymorphisms as biomarkers of risk for coronary heart disease. Biomark. Med..

[21] Liu Y., Wang J., Luo S., Zhan Y., Lu Q. (2020). The roles of PPARγ and its agonists in autoimmune diseases: A comprehensive review. J. Autoimmun..

[22] Grbić E., Peterlin A., Kunej T., Petrovič D. (2018). PPARγ gene and atherosclerosis: Genetic polymorphisms, epigenetics and therapeutic implications. Balk. J. Med. Genet..

[23] Dionisio K.L., Phillips K., Price P.S., Grulke C.M., Williams A., Biryol D., Hong T., Isaacs K.K. (2018). The Chemical and Products Database, a resource for exposure-relevant data on chemicals in consumer products. Sci. Data.

[24] EPA (2020). The Chemical and Products Database (CPDat) MySQL Data File. https://epa.figshare.com/articles/dataset/The_Chemical_and_Products_Database_CPDat_MySQL_Data_File/5352997.

[25] EPA (2021). Exploring ToxCast Data: Downloadable Data. https://www.epa.gov/chemical-research/exploring-toxcast-data-downloadable-data.

[26] Fang L., Zhang M., Li Y., Liu Y., Cui Q., Wang N. (2016). PPARgene: A Database of Experimentally Verified and Computationally Predicted PPAR Target Genes. PPAR Res..

[27] Judson R., Houck K., Martin M., Richard A.M., Knudsen T.B., Shah I., Little S., Wambaugh J., Setzer R.W., Kothiya P. (2016). Analysis of the Effects of Cell Stress and Cytotoxicity onIn Vitro Assay Activity Across a Diverse Chemical and Assay Space. Toxicol. Sci..

[28] Judson R.S., Magpantay F.M., Chickarmane V., Haskell C., Tania N., Taylor J., Xia M., Huang R., Rotroff D., Filer D.L. (2015). Integrated Model of Chemical Perturbations of a Biological Pathway Using 18In VitroHigh-Throughput Screening Assays for the Estrogen Receptor. Toxicol. Sci..

[29] Auerbach S., Filer D., Reif D., Walker V., Holloway A.C., Schlezinger J., Srinivasan S., Svoboda D., Judson R., Bucher J.R. (2016). Prioritizing Environmental Chemicals for Obesity and Diabetes Outcomes Research: A Screening Approach Using ToxCast™ High-Throughput Data. Environ. Health Perspect..

[30] Rager J.E., Ring C.L., Fry R.C., Suh M., Proctor D.M., Haws L.C., Harris M.A., Thompson C.M. (2017). High-Throughput Screening Data Interpretation in the Context of In Vivo Transcriptomic Responses to Oral Cr(VI) Exposure. Toxicol. Sci..

[31] Ring C., Sipes N.S., Hsieh J.-H., Carberry C., Koval L.E., Klaren W.D., Harris M.A., Auerbach S.S., Rager J.E. (2021). Predictive modeling of biological responses in the rat liver using in vitro Tox21 bioactivity: Benefits from high-throughput toxicokinetics. Comput. Toxicol..

[32] Todeschini R., Consonni V., Xiang H., Holliday J., Buscema P.M., Willett P. (2012). Similarity Coefficients for Binary Chemoinformatics Data: Overview and Extended Comparison Using Simulated and Real Data Sets. J. Chem. Inf. Model..

[33] Bajusz D., Rácz A., Héberger K. (2015). Why is Tanimoto index an appropriate choice for fingerprint-based similarity calculations?. J. Chemin..

[34] Helman G., Shah I., Williams A.J., Edwards J., Dunne J., Patlewicz G. (2019). Generalized Read-Across (GenRA): A workflow implemented into the EPA CompTox Chemicals Dashboard. ALTEX.

[35] Davis A.P., Murphy C.G., Saraceni-Richards C.A., Rosenstein M.C., Wiegers T.C., Hampton T.H., Mattingly C.J. (2009). GeneComps and ChemComps: A new CTD metric to identify genes and chemicals with shared toxicogenomic profiles. Bioinformation.

[36] Klaren W.D., Ring C., Harris M.A., Thompson C.M., Borghoff S., Sipes N.S., Hsieh J.-H., Auerbach S.S., Rager J.E. (2018). Identifying Attributes That InfluenceIn Vitro-to-In VivoConcordance by ComparingIn VitroTox21 Bioactivity VersusIn VivoDrugMatrix Transcriptomic Responses Across 130 Chemicals. Toxicol. Sci..

[37] Rager J.E., Clark J., Eaves L.A., Avula V., Niehoff N.M., Kim Y.H., Jaspers I., Gilmour M.I. (2021). Mixtures modeling identifies chemical inducers versus repressors of toxicity associated with wildfire smoke. Sci. Total Environ..

[38] Leydesdorff L. (2007). On the normalization and visualization of author co-citation data: Salton’s Cosine versus the Jaccard index. J. Am. Soc. Inf. Sci. Technol..

[39] Livak K.J., Schmittgen T.D. (2001). Analysis of Relative Gene Expression Data Using Real-Time Quantitative PCR and the 2^−ΔΔCT^ Method. Methods.

[40] Ryan K.R., Huang M.C., Ferguson S.S., Waidyanatha S., Ramaiahgari S., Rice J.R., Dunlap P.E., Auerbach S.S., Mutlu E., Cristy T. (2019). Evaluating Sufficient Similarity of Botanical Dietary Supplements: Combining Chemical and In Vitro Biological Data. Toxicol. Sci..

[41] Collins B.J., Kerns S.P., Aillon K., Mueller G., Rider C.V., Derose E.F., London R.E., Harnly J.M., Waidyanatha S. (2020). Comparison of phytochemical composition of Ginkgo biloba extracts using a combination of non-targeted and targeted analytical approaches. Anal. Bioanal. Chem..

[42] Kapraun D.F., Wambaugh J., Ring C., Tornero-Velez R., Setzer R.W. (2017). A Method for Identifying Prevalent Chemical Combinations in the U.S. Population. Environ. Health Perspect..

[43] Green A.J., Mohlenkamp M.J., Das J., Chaudhari M., Truong L., Tanguay R.L., Reif D.M. (2021). Leveraging high-throughput screening data, deep neural networks, and conditional generative adversarial networks to advance predictive toxicology. PLoS Comput. Biol..

[44] Wambaugh J.F., Wang A., Dionisio K.L., Frame A., Egeghy P., Judson R., Setzer R.W. (2014). High Throughput Heuristics for Prioritizing Human Exposure to Environmental Chemicals. Environ. Sci. Technol..

[45] Baker N., Knudsen T., Williams A.J. (2017). Abstract Sifter: A comprehensive front-end system to PubMed. F1000Research.

[46] EPA (2021). Benzyl Cinnamate. https://comptox.epa.gov/dashboard/chemical/details/DTXSID3041663.

[47] EPA (2021). 4-Hydroxybenzoic Acid Butyl Ester. 94-26-8 | DTXSID3020209. https://comptox.epa.gov/dashboard/chemical/details/DTXSID3020209.

[48] EPA (2021). Decanoic Acid. 334-48-5 | DTXSID9021554. https://comptox.epa.gov/dashboard/chemical/details/DTXSID9021554.

[49] Api A., Belsito D., Biserta S., Botelho D., Bruze M., Burton G., Buschmann J., Cancellieri M., Dagli M., Date M. (2020). RIFM fragrance ingredient safety assessment, decanoic acid, CAS Registry Number 334-48-5. Food Chem. Toxicol..

[50] EPA (2021). Eugenol. 97-53-0 | DTXSID9020617. https://comptox.epa.gov/dashboard/chemical/details/DTXSID9020617.

[51] EPA (2021). Sodium Dodecyl Sulfate. 151-21-3 | DTXSID1026031. https://comptox.epa.gov/dashboard/chemical/details/DTXSID1026031.

[52] Madrigal-Santillán E., Madrigal-Bujaidar E., Álvarez-González I., Sumaya-Martínez M.T., Gutiérrez-Salinas J., Bautista M., Morales-González Á., García-Luna Y., González-Rubio M., Aguilar-Faisal J.L. (2014). Review of natural products with hepatoprotective effects. World, J. Gastroenterol..

[53] Khanal T., Kim H.G., Jin S.W., Shim E., Han H.J., Noh K., Park S., Lee D.H., Kang W., Yeo H.K. (2012). Protective role of metabolism by intestinal microflora in butyl paraben-induced toxicity in HepG2 cell cultures. Toxicol. Lett..

[54] Rial S.A., Ravaut G., Malaret T.B., Bergeron K.-F., Mounier C. (2018). Hexanoic, Octanoic and Decanoic Acids Promote Basal and Insulin-Induced Phosphorylation of the Akt-mTOR Axis and a Balanced Lipid Metabolism in the HepG2 Hepatoma Cell Line. Molecules.

[55] Ulanowska M., Olas B. (2021). Biological Properties and Prospects for the Application of Eugenol—A Review. Int. J. Mol. Sci..

[56] Shah K.H., Verma R.J. (2011). Butyl p-hydroxybenzoic acid induces oxidative stress in mice liver—An in vivo study. Acta Pol. Pharm..

[57] Bondi C.A.M., Marks J.L., Wroblewski L.B., Raatikainen H.S., Lenox S.R., Gebhardt K.E. (2015). Human and Environmental Toxicity of Sodium Lauryl Sulfate (SLS): Evidence for Safe Use in Household Cleaning Products. Environ. Health Insights.

[58] Niraula T.P., Bhattarai A., Chatterjee S.K. (2014). Sodium dodecylsulphate: A very useful surfactant for scientific investigation. J. Knowl. Innov..

[59] OECD (2005). SIDS Initial Assessment Report for SIAM 5; Sodium Dodecyl Sulphate (CAS No: 151-21-3). https://hpvchemicals.oecd.org/ui/handler.axd?id=7ffa18c7-5c4c-4766-91d7-5967b67aeba3.

[60] NTP (2021). Chemical Effects in Biological Systems. https://cebs.niehs.nih.gov/cebs/.

[61] Davis A.P., Grondin C.J., Johnson R.J., Sciaky D., Wiegers J., Wiegers T.C., Mattingly C.J. (2020). Comparative Toxicogenomics Database (CTD): Update 2021. Nucleic Acids Res..

[62] Watford S., Pham L., Wignall J., Shin R., Martin M.T., Friedman K.P. (2019). ToxRefDB version 2.0: Improved utility for predictive and retrospective toxicology analyses. Reprod. Toxicol..

[63] Stanfield Z., Addington C.K., Dionisio K.L., Lyons D., Tornero-Velez R., Phillips K.A., Buckley T.J., Isaacs K.K. (2021). Mining of Consumer Product Ingredient and Purchasing Data to Identify Potential Chemical Coexposures. Environ. Health Perspect..

[64] CDC (2021). Fourth National Report on Human Exposure to Environmental Chemicals. https://www.cdc.gov/biomonitoring/pdf/fourthreport_updatedtables_feb2015.pdf.

[65] EPA (2021). Air Quality System (AQS). https://www.epa.gov/aqs.

[66] Ring C.L., Arnot J.A., Bennett D.H., Egeghy P.P., Fantke P., Huang L., Isaacs K.K., Jolliet O., Phillips K.A., Price P.S. (2018). Consensus Modeling of Median Chemical Intake for the U.S. Population Based on Predictions of Exposure Pathways. Environ. Sci. Technol..

[67] Wilkinson M.D., Dumontier M., Aalbersberg I.J., Appleton G., Axton M., Baak A., Blomberg N., Boiten J.W., da Silva Santos L.B., Bourne P.E. (2016). The FAIR Guiding Principles for scientific data management and stewardship. Sci. Data.

[68] Ragerlab (2022). Ragerlab Github. https://github.com/Ragerlab.

[69] Carberry C.K., Turla T., Koval L.E., Hartwell H., Fry R.C., Rager J.E. (2022). Dataset for Chemical Mixtures in Household Environments: In Silico Predictions and In Vitro Testing of Potential Joint Action on PPARg in Human Liver Cells. UNC Dataverse, V1. https://dataverse.unc.edu/dataset.xhtml?persistentId=10.15139/S3/31A4OD.

